# Innovative Methodology for Strengthening a Multidisciplinary Team Approach in Cities in Low- and Middle-Income Countries

**DOI:** 10.1200/GO.22.00149

**Published:** 2022-10-17

**Authors:** Vanessa Eaton, Angela Zambrano, Valeria Sanabria, Roberto Lopez, Ishmael Kyei, Rai Mra, Vanessa Sarchet, Megan Kremzier, Josep Borras, Thet Ko Aung, Rebecca Morton Doherty, Susan Henshall, Rolando Camacho

**Affiliations:** 1 American Society for Clinical Oncology, Alexandria, VA; 2 Fundación Valle Del Lili, Cali, Colombia; 3 Hospital Nacional de Itauguá del MoH—Hospital de Clínicas de la Universidad Nacional de Asunción—Centro Médico La Costa, Asunción, Paraguay; 4 Centro Médico La Costa, Asunción, Paraguay; 5 Komfo Anokye Teaching Hospital, Kumasi, Ghana; 6 Myanmar Medical Association, Yangon, Myanmar; 7 Department of Health, Catalonia, Spain; 8 City Cancer Challenge, Geneva, Switzerland

## Abstract

Delivering high-quality cancer care to patients through a multidisciplinary team (MDT) care approach remains a challenge, particularly in low- and middle-income countries characterized by fragmented health systems and limited human resources for cancer care. City Cancer Challenge (C/Can) is supporting cities in low- and middle-income countries as they work to improve access to equitable quality cancer care. C/Can has developed an innovative methodology to address the MDT gap, piloted in four cities—Asunciòn, Cali, Kumasi, and Yangon.

**METHODS:**

Collaborating with a network of partners, C/Can and ASCO have developed a package of technical cooperation support focusing on two priority areas that have emerged as core needs: first developing consensus-based, city-wide patient management guidelines for the most common cancers and second, building capacity for the implementation of MDTs in institutions providing cancer care in the city.

**RESULTS:**

The real-time application of C/Can's MDT approach in Cali and Asuncion underlined the importance of engaging the right stakeholders early on and embedding MDT guidelines in local and national regulatory frameworks to achieve their sustainable uptake. The results in Cali and Asuncion were essential for informing the process in Yangon, asserting the clear benefits of city-to-city knowledge exchange. Finally, the global COVID-19 pandemic prompted a rapid adaptation of the methodology from an in-person to virtual format; the unexpected success of the virtual program in Kumasi has led to its application in subsequent C/Can cities.

**CONCLUSION:**

The application of C/Can's methodology in this first set of cities has reinforced not only the importance of both resource appropriate guidelines and a highly trained health workforce but also the need for commitment to work across institutions and disciplines.

## INTRODUCTION

Quality cancer care has been defined as equitable, patient-centered, safe, effective, and timely care that is based on a collaborative approach and commitment to the continuous improvement of quality.^1^ As such, access to a broad, multidisciplinary scope of services, interventions, and specialized professionals with the capacity to respond to patients' medical and psychosocial needs^2^ is paramount. The coordinated planning, decision making, and information sharing between different health care providers that underpins this approach is defined as a Multidisciplinary Team (MDT) approach.^3,4^

**CONTEXT**

**Key Objective**
What are the key factors for successfully developing interventions aimed at strengthening a multidisciplinary team (MDT) care approach in cities in resource-constrained settings? The City Cancer Challenge Foundation and ASCO share their learnings and experience from project implementation in three cities in low- and middle-income countries.
**Knowledge Generated**
Leveraging local expertise and know-how, and early engagement of the right stakeholders, including government authorities, are essential factors for strengthening a MDT care approach in low- and middle-income countries. The value of peer-to-peer exchange supported by virtual communications and capacity development platforms is emphasized in this article.
**Relevance**
Multiorganizational collaboration across a diverse network of local cancer care professionals and global partners can accelerate efforts to strengthen a MDT care approach.

The organization of the delivery of cancer care as a whole directly influences the process and outcomes of patients with cancer.^5^ Although there is a paucity of robust studies on the direct impact of the MDT approach on patient outcomes, there is growing evidence for the application of team-based care for oncology in improving treatment efficiency and patient care for different tumor types.^3,6-8^ A MDT approach is characterized by four essential components: highly trained health care workers; clear, applicable treatment guidelines; a defined decision-making process for complex cases; and auxiliary administrative support for auditing and feedback. In addition, open communication among team members and a willingness to apply evidence-based guidelines are important elements of a successful multidisciplinary cancer care model. These must be coupled with a systematic approach to case management by nurses, ensuring patient navigation through the care pathway and encouraging patients to take an active role in their care.^9^

Delivering high-quality cancer care to patients remains a challenge in all settings, particularly in low- and middle-income countries where fragmented health systems and limited human resources for cancer care can be barriers to application of a multidisciplinary approach.

City Cancer Challenge (C/Can) supports cities in low- and middle-income countries as they work to improve access to equitable, quality cancer care. C/Can's City Engagement Process is a novel implementation framework, whereby local stakeholders lead a staged city-wide process over a 2- to 3-year period to assess needs and plan and execute locally adapted cancer care solutions.^10^ This process is supported by a global network of partners and experts, bringing together governments, the civil society, and the private sector. The results of needs assessments in a first set of cities^10^ identified a MDT approach as a recurring unmet need and priority, with specific efforts required to support its adoption at the local level.^11^

In response to this trend and on the basis of the priorities defined locally, C/Can and ASCO—in collaboration with their network of partners—have developed a package of technical cooperation support. The package addresses the limited available capacity at the local level to set up, operate, and monitor multidisciplinary cancer care in city institutions. This support focuses on two priority areas that have emerged as core needs: first, developing consensus-based, city-wide patient management guidelines for the most common cancers and second, building the capacity for the implementation of MDTs in institutions providing cancer care in the city.

The current article describes the innovative methodology and preliminary results of the delivery of the coordinated technical cooperation support implemented in Cali, Colombia; Asunción, Paraguay; Yangon, Myanmar; and Kumasi, Ghana. This article will also share the lessons learnt, which can be applied in the scale-up of this approach to other cities in low-resource contexts.

## METHODS

### Technical Cooperation Development

Specialists from the African Palliative Care Association (APCA), ASCO, the American Society of Clinical Pathology (ASCP), the International Atomic Energy Agency (IAEA), the International Society of Nursing in Cancer Care (ISNCC), the Latin American Association for Palliative Care (ALCP), the Oncology Nursing Society (ONS), and the WHO Collaborating Center for Training and Policy on Access to Pain Relief in Kerala, India, collaborated with C/Can on the following methodology. It supports the development of resource-appropriate guidelines for the management of patients with invasive cancer in the most common cancer sites and the sharing of knowledge and good practices for the implementation of MDTs in centers providing cancer care in the city.

The methodology aligns with C/Can's innovative city engagement process that employs an inclusive, multisector approach, bringing together local stakeholders to design and deploy context-driven solutions to cancer diagnosis and treatment.^10^ The resulting technical cooperation support was piloted in four cities: Cali, Colombia; Asunción, Paraguay; Yangon, Myanmar; and Kumasi, Ghana. The technical cooperation content was specifically tailored to the needs and requests of cities in each region.

The methodology outlined in this article was developed in an iterative process, incorporating early lessons and building on local experiences outlined in the city case studies (see the Results).

### Technical Cooperation Structure

The technical cooperation applies a uniform strategy on the basis of three components: local experts develop their own resource-appropriate guidelines tailored to local context and available resources in alignment with international best practices; existing local and regional expertise is leveraged to strengthen local capacities; and a network is developed for knowledge-sharing among cities.

#### Resource-appropriate guidelines.

A practical tool for city stakeholders, the *Methodology for City Projects on Access to Quality Cancer Care*, was developed to provide a step-by-step approach to producing resource-appropriate guidelines for the management of patients with invasive cancer in the most common cancer sites and a resolution on the implementation of MDTs in centers providing cancer care. The tool draws on previous guidance for resource-adapted guidelines for the treatment of breast and invasive cervical cancers developed for C/Can cities in cooperation with the Breast Health Global Initiative and Tata Memorial Hospital.^12,13^

**FIG 1:**
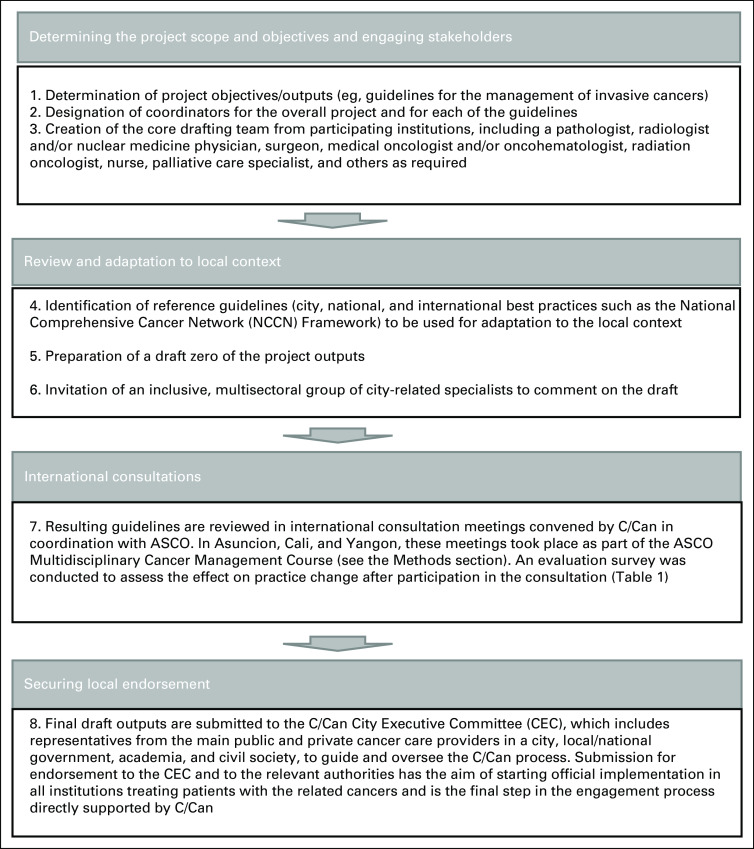
Steps for the development of resource-appropriate guidelines. C/Can, City Cancer Challenge; CEC, City Executive Committee.

A series of suggested steps guide cities along the process including (1) determining the project scope and objectives and engaging stakeholders, (2) review and adaptation of guidelines, (3) international consultations, and (4) securing local endorsement (shown in Fig 1).

The final step of the methodology pertains to preparing the implementation of the guidelines and where applicable a resolution on MDT. With a view to ensuring the sustainability of the project, C/Can expects relevant health authorities to approve the final drafts and set up and operationalize an implementation plan that includes a monitoring and evaluation system. In parallel, endorsement from professional associations is expected.

#### Capacity development and network.

Three cross-cutting opportunities are provided to local stakeholders to develop knowledge to work as a MDT, deepen knowledge on practical clinical cases pertaining to relevant cancer sites, and strengthen application of the guidelines. In combination, the three programs described here (TeleECHO, ASCO Multidisciplinary Cancer Management Course, and Scientific Visits) reinforce the importance of the MDT to deliver the best-quality cancer care for patients.

### TeleECHO

Once the final guidelines are submitted to the C/Can City Executive Committee (CEC; comprising key cancer care decision-makers from across sectors at city and national levels) for review, ASCO and C/Can coordinate a 6-month TeleECHO program consisting of 6 monthly virtual sessions. Participation is open to all city institutions. The sessions are 1 hour in length and consist of a short didactic expert presentation and a case developed by cancer care providers from the city. The aim of the sessions is to exchange knowledge between local and international experts and to discuss how the guidelines can be applied to different case scenarios.

### ASCO Multidisciplinary Cancer Management Course

The ASCO Multidisciplinary Cancer Management Course (MCMC) is a collaboration between ASCO and partners to deliver education and training in the multidisciplinary management of common cancer types. These courses consist of didactic presentations and multidisciplinary discussion of patients. The ASCO MCMC brings together international and national experts representing several disciplines including medical, surgical, and radiation oncology; pathology; imaging; oncology nursing; and palliative care. Societies represented include ASCO, ASCP, IAEA, ISNCC, ONS, palliative care associations, cancer centers, and local professionals. The MDT reviews MDT guidelines and serves as faculty. At the course, the coordinators of the guidelines drafting team present a general overview of the guidelines and an update on the status of their implementation. The discussion of case scenarios shows how the guidelines would be applied in a practice setting. Participants in this course are members of the drafting team and other relevant specialists. An evaluation survey was conducted to assess the effect on practice change after participation in the MCMC in Asuncion, Cali, and Yangon (Table 1). Surveys were administered on-site after the conclusion of each course and asked whether participants intended to make practice changes. One year later, an electronic survey was sent to participants asking if they had made actual practice changes on the basis of what they learned at the course.

**TABLE 1:**
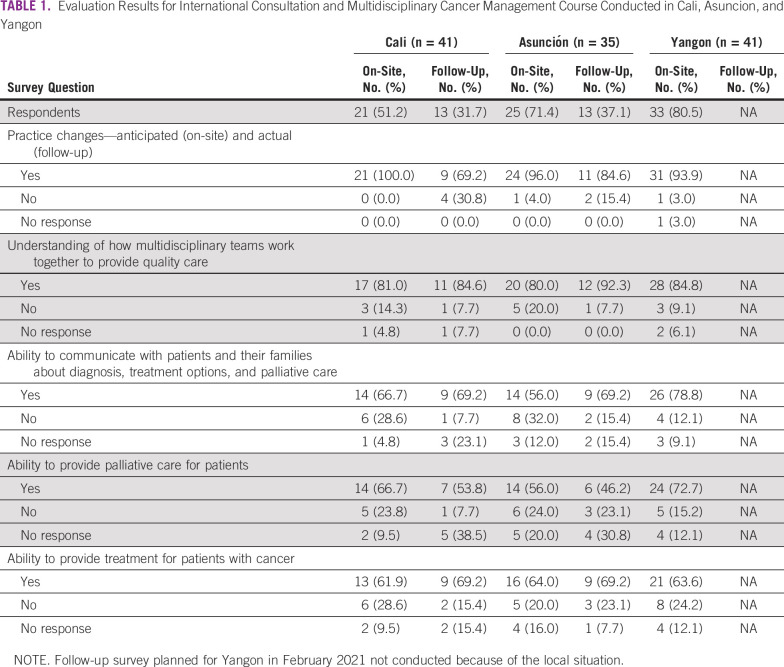
Evaluation Results for International Consultation and Multidisciplinary Cancer Management Course Conducted in Cali, Asuncion, and Yangon

### Scientific Visits

Scientific visits of city stakeholders to international reference centers provide cancer care professionals with practical work experience and insights into best practices in C/Can partner institutions through an immersive, peer-to-peer exchange experience. Locally designated groups of professionals from Asuncion, Cali, and Kumasi (total 42 health professionals) representative of the most relevant cancer specialties (pathology, medical imaging, surgery, medical oncology, radiation oncology, nursing, and supportive/palliative care providers) participated in visits between 2019 and 2021 to three leading cancer care centers: Hospital del Mar, Barcelona, Spain; Instituto Catalan de Oncologia, Barcelona, Spain; and Mayo Clinic, Rochester, NY. The visits were tailored to support learning best practice in the multidisciplinary approach, to build long-term professional relationships with experts from the reference centers, and to encourage knowledge exchange with local peers on return to the cities.

## RESULTS

The following three case studies describe the application of the methodology in Cali, Asuncion, Yangon, and Kumasi, illustrating the core components and how these are being leveraged to strengthen, scale, and sustain the adoption of a MDT approach in the cities.

### Case Study 1: Ensuring Sustainable Uptake of Resource-Adapted Guidelines in Cali and Asuncion

In Cali, Colombia, a city-wide assessment of cancer care needs was performed from May to December 2017.^10^ This process highlighted the challenge of patients with cancer receiving fragmented care, disparities in treatment across different institutions in Cali, and the need for standardized, integrated cancer patient management.

In response, the C/Can CEC approved a project to develop guidelines for managing patients with the most common cancers, starting with cervical and breast cancer.

Stakeholders in Asunción, Paraguay, prioritized a similar project on the basis of a needs assessment led by 200 professionals from 16 cancer care providers from July to December 2017.

In both cities, C/Can's methodology was applied, with guidelines locally developed by core drafting teams for the respective cancer sites. Set up with C/Can support, these multidisciplinary drafting teams consisted of pathologists, medical imaging specialists, surgeons, medical oncologists, radiation oncologists, palliative and supportive care specialists, and nurses. Expert input was also provided by ASCO, ASCP, IAEA, and ONS during a 2-day MCMC. The MCMC that took place in Cali in July 2019 was attended by 41 professionals from 18 institutions and was followed up with a round of virtual international consultations with ASCO (Table 1). Similarly, the MCMC for Asuncion took place in August 2019 with participation from 35 cancer care specialists. In addition, a multidisciplinary group of 28 health professionals from Cali and Asuncion participated in a scientific visit to Hospital del Mar and Instituto Catalan de Oncologia, Barcelona, Spain, in November 2019.

The iterative and inclusive process of drafting the guidelines with guidance from C/Can and ASCO equipped the teams in both cities with the concrete skills and know-how to develop and implement resource-adapted guidelines tailored to the local context. In Cali, leveraging this knowledge, local stakeholders decided to expand the project scope to include three additional cancer sites—colorectal cancer, prostate cancer, and pediatric leukemia. With support from a local partner organization, ProPacifico (a local nonprofit foundation to which C/Can transitioned oversight of Cali projects in November 2019), the three additional guidelines were developed between January and October 2020 using the same methodology.

To help ensure the sustainable uptake of these guidelines, local stakeholders clearly identified the need for high-level political commitment and endorsement by regional and national governments. Final draft guidelines were submitted to the C/Can CEC, which included representation from the Ministry of Health and Regional Health Secretary, in November 2020. As a result, a first-time collaboration agreement for the implementation of cancer management guidelines for the city's five prioritized cancers was formally signed in July 2021 by the Secretary of Health of Valle del Cauca, the Secretary of Health of Cali, and Cali's leading hospitals and insurance agencies. Four of the five guidelines—breast,^14^ cervical,^15^ prostate,^16^ and colorectal^17^—have since been published by ProPacifico, with the guideline for pediatric cancers due to be finalized in the second quarter of 2022.

In Asuncion, local stakeholders emphasized the importance of national endorsement for sustainable implementation, with guidelines submitted to the Ministry of Health of Paraguay and subsequently adopted at a national level at the end of 2021. Consequently, and for the first time in the country, the breast cancer guidelines are now formally endorsed throughout Paraguay's public hospitals.^18^ Cervical cancer guidelines have been finalized and are due to be submitted to the Ministry of Health in the second quarter of 2022. The widespread adoption of a MDT approach is being reinforced by a resolution issued by the Ministry of Health, requiring institutions across the country that manage patients with cancer to set up multidisciplinary tumor committees.

### Case Study 2: Sharing Knowledge through City-to-City Exchange in the C/Can Network

With the aim of fostering knowledge exchange and peer learning across cities and regions, the experience of Asuncion was used to inform the approach taken in Yangon.

A needs assessment was implemented from October 2017 to January 2018 with the involvement of 172 health professionals from 20 public and private institutions and 260 patients and caregivers in Yangon. The C/Can CEC prioritized the need to develop a MDT resolution and resource-adapted guidelines for the management of breast and invasive cervical cancers.

Guideline development followed C/Can's methodology with local MDTs created to review existing national and international guidelines and to consult with international experts nominated by C/Can and its partners including ASCO, ASCP, IAEA, ISNCC, and the WHO Collaborative Center on Community Participation in Palliative Care and Long-term Care. To facilitate the sharing of relevant experience from Asuncion, in February 2020, the MCMC course attended by 41 cancer care professionals in Yangon was supported by the coordinator for the drafting of the MDT resolution in Asuncion.

On the basis of recommendations from the MCMC and with the support of international experts, the guidelines for breast and cervical cancer were finalized and endorsed by the C/Can CEC and the Ministry of Health and Sports in November 2020.

### Case Study 3: Strengthening Local Capacity by Leveraging Virtual Platforms

In Kumasi, Ghana, a city-wide needs assessment involving 257 health care professionals from 38 cancer care institutions was performed from February to August 2018. On the basis of the findings, a project was designed to make MDTs available as standard practice for the management of patients with breast and cervical cancers. It included the drafting of standardized treatment guidelines, development of a norm to establish and regulate MDTs, and creation of a digital platform for virtual case discussion.

A consultation was scheduled to take place in April 2020 to support the locally led drafting teams and facilitate a review of the guidelines with international expert input. Because of the global pandemic and resulting travel restrictions, C/Can and ASCO were challenged to rapidly adapt the aforementioned methodology and coordinate a Virtual International Consultation. Experts were nominated by APCA, ASCO, ASCP, IAEA, and ISNCC to join a series of calls between August 2020 and February 2021 with the local team to review and agree on the final draft guidelines. In addition, 14 health care professionals from Kumasi participated in a scientific visit to Mayo Clinic, Rochester, NY, in December 2021. The guidelines were approved by the Ministry of Health in July 2021 and submitted to the C/Can CEC for endorsement in March 2022.

Moving from an in-person to virtual consultation model resulted in a more iterative drafting process, with additional time for review and integration of comments before each call. Proving to be a more efficient way for local and international experts to collaborate on guideline development, this approach is now being adopted in other C/Can cities, including Porto Alegre, Brazil, and Tbilisi, Georgia.

## DISCUSSION

As an accompaniment to the growing evidence of the importance of a multidisciplinary approach in improving the quality of cancer care, the work described here reinforces the necessity for a structured methodology to enable this approach to be adopted in low-resource settings.

Although the methodology remains iterative, there are already common key enablers emerging. First is support for local health professionals to acquire know-how on drafting the documents. In addition, the creation of a network of international partners and experts that can be drawn on for alignment with international best practices, but also to support drafting new guidelines on other cancer sites, as was the case in Cali, Colombia, is a key enabler for success.

By creating ownership and interest in expanding the MDT approach to other common cancers, the methodology therefore encourages long-term engagement and sustainability of the approach. It also fosters a culture of collaboration and willingness to agree on evidence-based clinical decisions among the different specialties of the MDT that will ultimately facilitate implementation at the institutional level. This was supported by the positive response to the impact on practice changes—both intended (on-site) and actual (follow-up), observed in Cali, Asuncion, and Yangon.

Another key learning is the importance of engaging the right stakeholders. In Cali, Colombia, ProPacifico identified payers and insurers as a critical stakeholder group that should be engaged in the process. They were invited to participate in the final drafting stage of treatment guidelines to provide a perspective on how funding is managed and patients' needs in this regard. Local stakeholders felt that this improved the drafting team's understanding of patient needs and contributed to the successful endorsement of the guidelines.

In all four pilot cities, it was imperative to achieve early engagement of relevant government authorities, which would later drive the necessary policy processes to formalize and disseminate guidelines. The C/Can methodology recognizes the endorsement of final documents by the C/Can CEC and submission to local, regional, or national authorities for approval and implementation as a critical final step in the methodology. However, experience in the first cities has further emphasized that incorporating a MDT approach and guideline use in local or national regulations and norms will provide an additional weight to the documents. This is the case in Asuncion, where a draft ministerial resolution is introducing the MDT approach including the guidelines as standard practice for each institution in the city.

Prioritizing and leveraging existing local expertise and know-how is a central premise of C/Can's approach to technical cooperation support,^10^ which on the basis of the experience in this first set of cities can be augmented by international expert consultation (Table 1). In Cali, in particular, local stakeholders reported that working with international experts to review the guidelines not only ensured alignment with international standards but also provided the documents with independent feedback and thus guaranteed a high level of credibility and quality while maintaining local ownership and leadership of the process.

Finally, C/Can's experience in working across cities and regions has also demonstrated the value and high interest in city-to-city exchange as another mechanism to share expertise. As cities across the C/Can network prioritize the development of context-specific guidelines, opportunities for information sharing and exchange of both the development process and the content are anticipated to further accelerate and enhance the process in future C/Can cities.

Accelerated by the pandemic, C/Can has focused on strengthening its city-to-city network supported by virtual communications and capacity development platforms including the TeleECHO program. In Kumasi—despite the change to a virtual format—more than 100 attendees in total participated in the six TeleECHO sessions held on the breast and cervical cancer guidelines.

In conclusion, the application of C/Can's technical cooperation support for a MDT approach in this first set of cities has reinforced the importance of not only practical components, including resource appropriate guidelines and a highly trained health workforce, but also the commitment and willingness to work across institutions and disciplines.

A key short-term outcome reported by local stakeholders in all four cities was the strengthened communication between clinicians across disciplines and institutions around the common goal of improving quality. The evaluation survey responses also indicate the intent for practice change as a result of the integrated technical cooperation support. Although it is premature to conclude a positive impact on the adoption of MDT care at an institutional level at this point in time, a critical next step will be to monitor and further evaluate the adoption of the approach at the local level.
